# Topical Polyethylene Glycol as a Novel Chemopreventive Agent for Oral Cancer via Targeting of Epidermal Growth Factor Response

**DOI:** 10.1371/journal.pone.0038047

**Published:** 2012-06-04

**Authors:** Ramesh K. Wali, Dhananjay P. Kunte, Mart De La Cruz, Ashish K. Tiwari, Jeffrey Brasky, Christopher R. Weber, Tina P. Gibson, Amir Patel, Suzana D. Savkovic, Bruce E. Brockstein, Hemant K. Roy

**Affiliations:** 1 Department of Medicine, NorthShore University Healthsystem, Evanston, Illinois, United States of America; 2 Department of Pathology, University of Chicago, Chicago, Illinois, United States of America; IIT Research Institute, United States of America

## Abstract

Head and neck squamous cell carcinoma (HNSCC) is a major cause of morbidity and mortality underscoring the need for safe and effective chemopreventive strategies. Targeting epidermal growth factor receptor (EGFR) is attractive in that it is an early critical event in HNSCC pathogenesis. However, current agents lack efficacy or have unacceptable toxicity. Several groups have demonstrated that the over-the-counter medication, polyethylene glycol (PEG) has remarkable chemopreventive efficacy against colon carcinogenesis. Importantly, we reported that this effect is mediated through EGFR internalization/degradation. In the current study, we investigated the chemopreventive efficacy of this agent against HNSCC, using both the well validated animal model 4-NQO (4-nitroquinoline 1-oxide) rat model and cell culture with the human HNSCC cell line SCC-25. We demonstrated that daily topical application of 10% PEG-8000 in the oral cavity (tongue and cavity wall) post 4NQO initiation resulted in a significant reduction in tumor burden (both, tumor size and tumors/tumor bearing rat) without any evidence of toxicity. Immunohistochemical studies depicted decreased proliferation (number of Ki67-positive cells) and reduced expression of EGFR and its downstream effectors cyclin D1 in the tongue mucosa of 4NQO-rats treated with PEG. We showed that EGFR was also markedly downregulated in SCC-25 cells by PEG-8000 with a concomitant induction of G1-S phase cell-cycle arrest, which was potentially mediated through upregulated p21^cip1/waf1^. In conclusion, we demonstrate, for the first time, that PEG has promising efficacy and safety as a chemopreventive efficacy against oral carcinogenesis.

## Introduction

Squamous cell carcinoma of the head and neck region (HNSCC) is the sixth most prevalent cancer worldwide, accounting for 3% of all cancers [1]. In 2010, in the US alone there were an estimated 49,000 new HNSCC cases and 11,500 HNSCC related-deaths [2]. Importantly, these numbers do not take into account severe morbidity from the facial disfigurement and aerodigestive dysfunction associated with surgery/radiotherapy. Prevention of this malignancy, therefore, represents a major healthcare imperative. Modifications of certain life-style risk factors would be ideal but difficult to achieve despite major public health efforts against tobacco use (both smoked and chewed), betel nut chewing, alcohol consumption and HPV (infection) status.

Therefore, interest has focused on chemoprevention given that the at-risk groups are well defined for primary prevention efforts; those with early neoplastic transformation (oral leukoplakia) which can be identified by a standard physical exam. An equally important application would be secondary chemoprevention (preventing second primaries HNSCC in patients with a previous history of cancer).

It has been noted that even after successful tumor resection (histopathologically clear margins); ∼20% patients may still have recurrence of HNSCC at a different site (about 2% per year) [3]. This has largely been attributed to “field cancerization” et [4]. Indeed, classic studies have suggested that several mutational events in the microscopically normal mucosa can be predictive of recurrent HNSCC and overall survival [5]. This “condemned mucosa” concept is robust not only for prevention of recurrence (secondary prevention) but also presents a potential target for primary chemoprevention (patients without cancer but having premalignant lesions).

Thus, finding molecular targets in the premalignant mucosa has been an overarching theme in HNSCC prevention with epidermal growth factor receptor (EGFR) receiving some attention. EGFR is a critical early event in HNSCC and is overexpressed in >80% of HNSCC. EGFR overexpression and increased copy number in oral premalignant lesions is an excellent predictor of the risk of progression to HNSCC [6]. In addition, EGFR overexpression has been found in histologically normal mucosa from HNSCC patients indicating that altered EGFR signaling contributes to the field cancerization seen in these patients [7]. Importantly, targeting EGFR is a stalwart for anti-HNSCC therapies underscoring the importance of this pathway. However, as with most other molecular-targeted drugs, the major issues concerning the use of anti-EGFR agents (monoclonal antibodies, small molecule inhibitors, etc) for chemoprevention are their high costs for prolonged use and associated toxicity, especially given that the majority of patients offered chemopreventive agents do not have cancer. Therefore, finding an inexpensive, well tolerated mechanism to target EGFR in oral mucosa would be a major step forward in HNSCC chemoprevention effort.

Our group has been exploring the over-the-counter laxative polyethylene glycol (PEG) for its remarkable potency at downregulating EGFR and thus providing a potential explanation for its colon cancer chemopreventive efficacy (documented by several groups in a number of pre-clinical models) [8], [9], [10], [11], [12], [13]. From a mechanistic point of view, we observed that PEG resulted in rapid internalization of membrane bound EGFR with concomitant proteosomal degradation. This leads to decreased cyclin D1 and SNAIL (implicated in both colorectal cancer and HNSCC) thus transducing the anti-neoplastic effects of PEG [13].

We therefore hypothesized that topical PEG may be an effective chemopreventive agent against HNSCC. For these studies we used a well-validated carcinogen, 4-Nitroquinoline 1-oxide (4-NQO)-treated rat model of HNSCC and squamous cancer cell line, SCC25 cells. Given the concern that PEG may confound the effect with a direct carcinogen-oral mucosal interaction, we used a post-initiation design using tumor size and multiplicity as our primary endpoints and the well validated intermediate biomarkers of proliferation as a secondary endpoint. We, herein for the first time, demonstrate that daily topical oral application of PEG-8000 for a short interval significantly decreased the oral tumor burden (both tumor size and number).

## Materials and Methods

### Animals Studies and Tumor Induction

All animal protocols were reviewed and approved by the Institutional Animal Care and Use Committee of NorthShore University HealthSystem (IACUC Assurance # A3444-01; protocol # 07-230). Twenty-four male Fisher rats (F 344; 150–200 g; Harlan, Indianapolis, IN) were housed in a climate controlled environment (25°C temperature, 60% humidity and a 12 h light/day cycle). Sixteen animals were provided *ad libitum* rat chow and drinking water supplemented with 4-nitroquinoline 1-oxide (4NQO; 20 ppm; Sigma Chemicals). Freshly made 4NQO supplemented water was dispensed to rats in light opaque bottles that were replenished two times a week. The remaining 8 rats were provided clean drinking water (control group). After 14 weeks, the 4NQO supplemented water was replaced with regular water and the rats were randomized into two treatment groups. The first group (8 rats) received a daily topical application of 10% (W/V) PEG-8000 (the dosage/formulation effective in colon cancer chemoprevention) by painting the buccal floor/roof of the rat oral cavity using a sable brush (#4) for up to 3–4 minutes. For these treatments, the rats were mildly sedated in a well regulated isoflurane/oxygen anesthetic chamber. The second group (8 rats), serving as the control, was sham painted with the brush dipped in saline only. This regimen was continued for 14 additional weeks. At necropsy, rat tongues were excised, and subjected to macroscopic tumor assessment. The tongue sections were sliced, formalin fixed, paraffin embedded, sectioned and subjected to histological and immunohistochemical processing.

### Tumor Count and Volume

The dissected tongues were examined for the presence of overt tumors. Total number of tumors (>0.2 cm) on each tongue were counted and the tumor volume (size) was measured according to the formula width × length × height × π/6 [14]. Histological evaluations for the presence of epithelial atypia and dysplasia in the uninvolved tongue tissue were performed after staining with hematoxylin and eosin.

### Immunohistochemical (IHC) Analysis

The tongue sections were subjected to IHC analysis to determine the effect of PEG on the expression of proliferation markers Ki67, EGFR and Cyclin D1. Four micron paraffin-embedded sections were mounted on Superfrost^+^ slides (Vector Laboratories, Burlingame, CA) and deparaffinized first by baking at 55–60°C for 1 hour and then subjecting to two 5 minute washes in xylene. The tissue sections were then hydrated in graded series of ethanol rinses. The epitope retrieval was performed by subjecting the tissue slides to pressure microwaving (NordicWare, Minneapolis, MN) in antigen unmasking solution (Vector Laboratories). Endogenous peroxide activity was quenched by treating with 3% H_2_O_2_ in methanol for 10 min and the nonspecific binding was blocked by incubating the tissue sections with 5% horse serum for 1 hour at room temperature. Sections were then incubated at 4°C for 4–6 hours with primary antibodies [anti-Ki67 (1∶250; AbCam, Cambridge, MA), anti-EGFR (1∶200; Santa Cruz Biotechnology, Santa Cruz, CA) and anti-Cyclin D1 (1∶100; Cell Signaling Technology, Danvers, MA)], followed by appropriate biotinylated secondary antibodies. The antigen-antibody complexes were detected with the Vectastatin Elite ABC kit (Vector Laboratories) using 3, 3′ –diaminobenzidine (DAB) as chromagen (Vector Laboratories). For negative controls, sections were processed in the absence of the primary antibodies. Specimens were counterstained in Gill’s hematoxylin solution and the blue color stabilized by a 20 second wash in saturated lithium carbonate (1 g/100 ml). IHC was scored by the pathologist (CW) with no prior knowledge of the treatment plan. A semi-quantitative scale was used to evaluate immunoreactivity of basal squamous epithelial cells. The extent of staining was graded and scored as 0, negative staining; 1+, <10% reactivity, 2+10–50% reactive, and 3+ for >50% positive reactivity [15].

### Cell Culture

SCC-25 cells were obtained from American Type Tissue Culture (ATCC), Rockville, MD. These are poorly differentiated squamous cells obtained from human tongue. The identity and quality of these cells were authenticated by ATCC. The cells were cultured in DMEM/F-12 media (containing 2.5 mM L-glutamine, 15 mM HEPES, 0.5 mM sodium pyruvate, and 1200 mg/L sodium bicarbonate) supplemented with 400 ng/ml of hydrocortisone (Sigma/Aldrich), 10% FBS (ATCC), and 0.5% Pen/Strep (ATCC). To assess the effect of PEG, these cells were treated with either PEG-8000 or vehicle (PBS) for 24 h. Cells were then harvested and subjected to western blot and flow cytometric analyses.

### Cell Proliferation Assay

Cell number was assessed by WST-1 (4-[3-(4-iodophenyl)-2-(4-nitrophenyl)-2H-5-tetrazolio]-1, 3-benzene disulfonate) assay according to the manufacturer’s instructions (Roche Diagnostics, Indianapolis, IN). Briefly, SCC-25 cells were grown in 96 well plates in a final volume of 100 µl and then incubated with 10 µl of the WST-1 reagent at 37°C for 30 min in a humidified 5% CO_2_ incubator. Conversion of tetrazolium salt into formazan was determined spectrophotometrically at 440 nm absorbance (Molecular Devices, Sunnyvale, CA).

### Cell Cycle Analysis

SCC-25 cells were incubated with vehicle (phosphate buffered saline; PBS) or 10% PEG in a humidified 5% CO_2_ incubator at 37°C for 24 h. The cells were subsequently washed in PBS/bovine serum albumin (BSA), trypsinized, resuspended in fresh PBS/BSA and fixed in 70% ethanol at -20°C for 30 min. After 2 washes, the cells were incubated for 3 h (at room temperature) in PBS/BSA solution containing propidium iodide (PI; 40-µg/ml, Sigma) and RNase A (200 µg/ml; Sigma). The cells were subjected to DNA content measurement using flow cytometry (Becton Dickinson Labware). The data was expressed as percentage of cells in G_o_–G_1_ through G_2_-M populations and CellQuest 3.1 software program was used for the development of DNA content frequency histograms.

### Western Blot Analysis

Western blotting was applied using standard techniques. Briefly, 30 µg protein was subjected to SDS-PAGE, transferred to polyvinylidene difluoride membranes (Amersham Pharmacia, Piscataway, NJ), blocked with 5% blotto and probed with specific antibodies including proliferating cell nuclear antigen (PCNA), P21^cip1/waf1^, Cyclin D1, epidermal growth factor receptor (EGFR). Xerograms were developed with enhanced chemiluminescence (Santa Cruz Biotechnology). Images were acquired via UVP Bio-imaging Systems and analyzed using Labworks 4.6 software. Uniformity in protein loading was achieved by normalization after probing membranes with anti-β-actin (1∶1000).

### Statistical Methods

Values were expressed as mean + SE as indicated. Quantitative densitometry values were compared by paired Student’s test. Differences with p<0.05 were considered statistically significant.

## Results

### PEG Inhibits 4NQO-induced Oral Tumor Initiation and Progression

**Figure 1:**
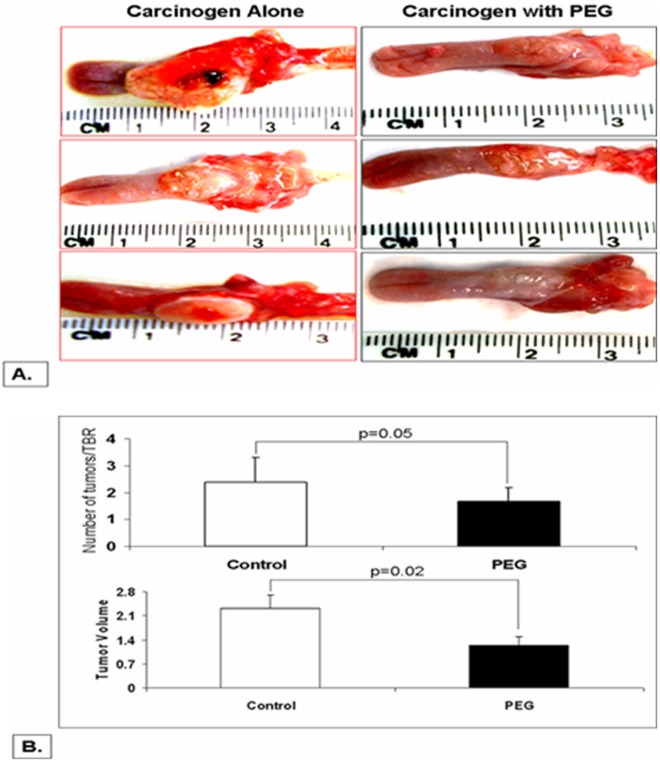
Effect of topical oral application of PEG-8000 on the initiation and progression of 4NQO-induced oral cancer –Fisher rats were provided 4NQO (20 ppm) in drinking water for 14 weeks before switching to regular water and randomizing into two groups. The first group received a daily (3–4 minute) topical application of 10% PEG-8000 via oral painting and the second group was sham painted (PEG-control group). This regimen was continued for 14 additional weeks before euthanization. The rats were euthanized after 14 weeks and the oral cavity subjected to macroscopic tumor assessment of total tumors (≥0.2 cm). As shown, 4NQO-treated rats developed multiple large tumors in the oral cavity mostly originating from tongue and few from the wall of the oral cavity. PEG reduced the overall tumor number (tumors/tumor bearing rat) (p = 0.05) and the growth of tumors (tumor volume) compared to their age-matched counterparts (p = 0.02). The tumor volume (size) was measured according to the formula width × length × height × π/6.

The rat 4NQO-induced oral carcinogenesis model is a routinely used model for HNSCC chemoprevention studies which shares a number of characteristics with human HNSCC carcinogenesis, in terms of multi-step molecular events and sequential changes in the histopathological features of the oral cavity mucosa [16], [17]. One of the other advantages of this model is the relatively high specificity toward head and neck tumors, as well as minimal debilitating effect on the general health of the rats. As shown in Figure 1 (A), at necropsy (14 weeks after the end of carcinogen treatment), 4NQO-treated rats developed multiple large tumors in the oral cavity, mostly originating from tongue and some from the oropharyngeal mucosa. Administration of PEG-8000 for 14 weeks after completing 4NQO treatment demonstrated two significant changes: 1) PEG-8000 treated rats developed significantly smaller tumors (tumor volume) than their age- and 4NQO treatment-matched counterparts (∼58% decrease; p = 0.02); 2) the overall tumor number (tumors/tumor bearing rat) in PEG-8000 treated rats was also lower than age- and 4NQO treatment-matched rats which did not receive PEG-8000 at any point (∼25% decrease; p = 0.05) (Figure 1B). These results imply that PEG-8000 application can effectively reduce formation of new tumors (inhibition of initiation) as well as halt the progression of pre-existing tumors toward advanced HNSCC (inhibition of progression).

### PEG Suppresses Premalignant Epithelial Hyperproliferation

**Figure 2:**
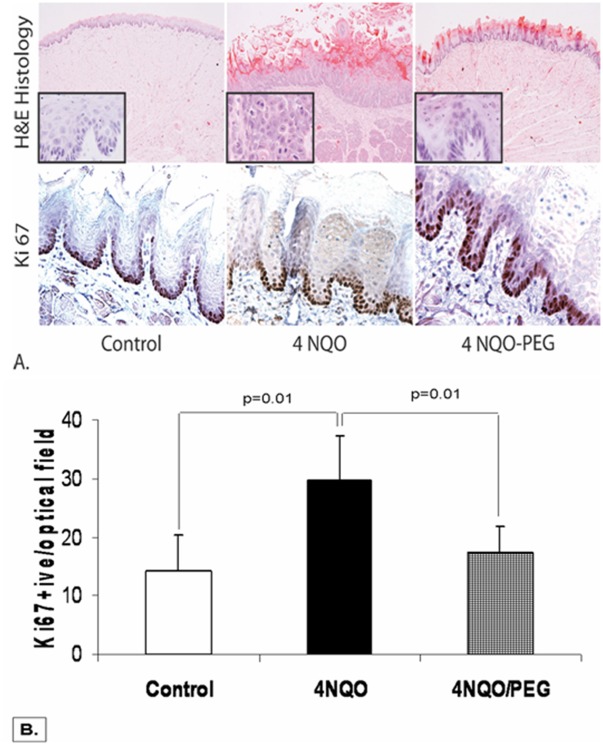
Effect of topical oral application of PEG-8000 on the premalignant epithelial hyperproliferation – To evaluate histopathological grading of the oral/tongue tissue, the formalin fixed sections were paraffin embedded, sectioned and subjected to H&E staining. As shown (top Panel; Figure 2A), 4NQO-treated rat sections revealed many localized regions of mild to moderate epithelial dysplasia in morphologically normal appearing mucosa that was normalized by PEG [higher magnification (63×; cropped image) insets demonstrate the presence of mitotic figures in the 4 NQO-treated group alone]. To further study the effects of PEG on the mucosal hyper-proliferation, we performed the immunohistochemical analysis of nuclear antigen Ki67, a well-defined marker of proliferation. A total of 1000 epithelial cells were evaluated from 6–7 fields. As shown (bottom Panel; Figure 2A–B), 4NQO-treatment increased the proliferation in morphologically normal tongue mucosa as depicted by increased number of Ki67-labelled epithelial cells/per optical field compared to age matched carcinogen free controls. (p<0.01). Topical application of PEG on the other hand dramatically reduced the number of Ki67-labelled epithelial cells/per optical field (p<0.01).

A precise temporal and spatial regulation of the diffuse cellular proliferation is an important characteristic of early-stage HNSCC carcinogenesis, and serves as an important tool for assessing chemopreventive effectiveness of agents. In 4NQO-treated rat model of HNSCC carcinogenesis, generalized cellular hyperproliferation in the tongue epithelium has previously been reported [18]. Microscopic evaluation of the mucosa in 4NQO-treated rats revealed many localized regions of mild to moderate epithelial dysplasia in morphologically normal tongue and oral mucosa that were normalized by PEG (Figure 2A). To further study the effects of PEG-8000 application on proliferation in the tongue/oral mucosa of 4NQO-treated rats, we examined the immunohistochemical expression of nuclear antigen Ki67, a well-defined marker of proliferation. For this, a total of 1000 epithelial cells were evaluated in 6–7 fields at 400 × magnification and all the values were used for the labeling indices. As shown in Figure 2A and B, 4NQO-treatment significantly increased the proliferation in morphologically normal tongue mucosa as depicted (number of Ki67-labelled epithelial cells/per optical field: 30+8 in 4NQO-treatment group vs. 14+6 in matched controls; p<0.01). Topical application of PEG-8000 dramatically normalized proliferation indices in 4NQO-treated rats (number of Ki67-labelled epithelial cells/per optical field: 30+8 in rats treated with 4NQO-alone vs. 17+4 in 4NQO-treated rats treated with PEG-8000; ∼43% reduction; p<0.01). In the mucosal epithelium of 4NQO-treated rats, as opposed to normal squamous epithelium where proliferation is limited to the basal compartment, the proliferation zone extended to the supra-basal compartment of the stratified squamous tongue epithelium. However, PEG-8000 application contained the proliferative zone back to the basal compartment of the stratified squamous epithelia, indicating strong anti-proliferative effects of this topical agent.

### PEG Downregulates Tongue Mucosal EGFR and Cyclin D1 Expression in 4 NQO-treated Rats

**Figure 3:**
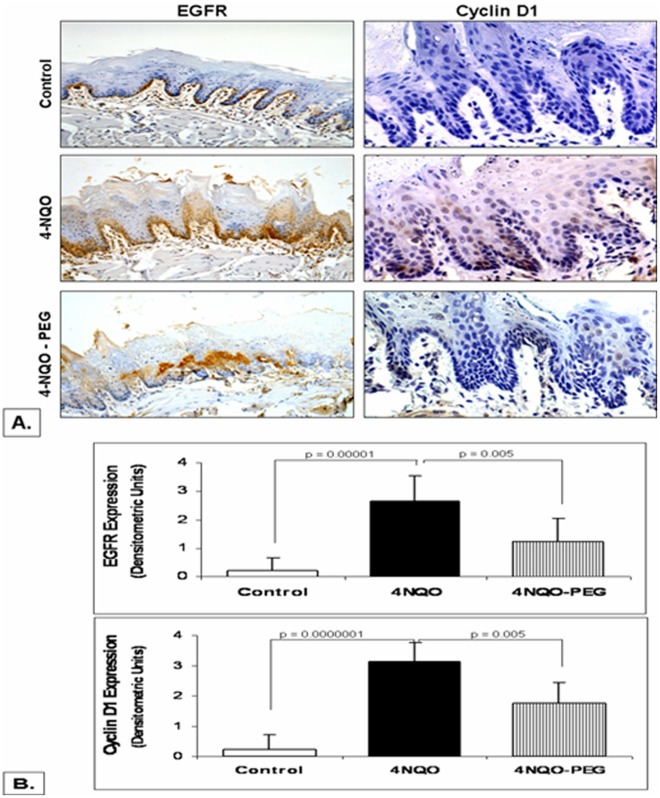
Effect of PEG-8000 on tongue mucosal EGFR and cyclin D1 expression in 4 NQO-treated rats –The tongue sections were subjected to immunohistochemical analyses to assess changes in EGFR and Cyclin D1 expressions. As shown (left Panel - Figure 3A and 3B), baseline EGFR expression in the tongue mucosa of 4NQO-rats was higher than that of control rats (p<0.00001). Topical application of PEG however, caused a significant decline in the expression of EGFR (p<0.005). Furthermore, topical PEG application to 4-NQO rats caused similar effects on the expression of Cyclin D1, one of the downstream effectors of EGFR (right Panel - Figure 3A and 3B).

EGFR overexpression in the premalignant head and neck lesions is correlated with the increased risk of progression to HNSCC and poor survival [19], [20]. Modulating EGFR expression can therefore serve as the key element of a chemopreventive strategy. Several groups, including ours have previously shown that anti-proliferative and chemopreventive effects of PEG against colorectal carcinogenesis are EGFR-mediated, as administration of PEG-gavages in carcinogen treated rats significantly reduced proliferation in colonic mucosa through downregulation of EGFR expression [13]. We therefore, investigated if a similar mechanism was implicated in the chemopreventive actions of topical PEG-8000 against HNSCC. The tongue sections were formalin fixed and examined by immunostaining to assess changes in the expression levels of EGFR. As shown in Figure 3A and B, topical application of PEG-8000 significantly lowered the intensity as well as the number of areas overexpressing EGFR in 4NQO-treated rats where baseline EGFR expression was much higher than the tongue/oral mucosa of healthy control rats. This implicates downregulation of EGFR as one of the potential mechanisms for PEG-induced chemoprevention of HNSCC. We further demonstrated that PEG significantly reduced the expression of Cyclin D1, a downstream effector of EGFR that is overexpressed in HNSCC [21] and involved in causing resistance to therapeutic drugs such as cisplatin [22] and gefitinib [23].

### PEG Inhibits Cellular Proliferation and Induces Cell Cycle Arrest in SCC-25 Cells

**Figure 4:**
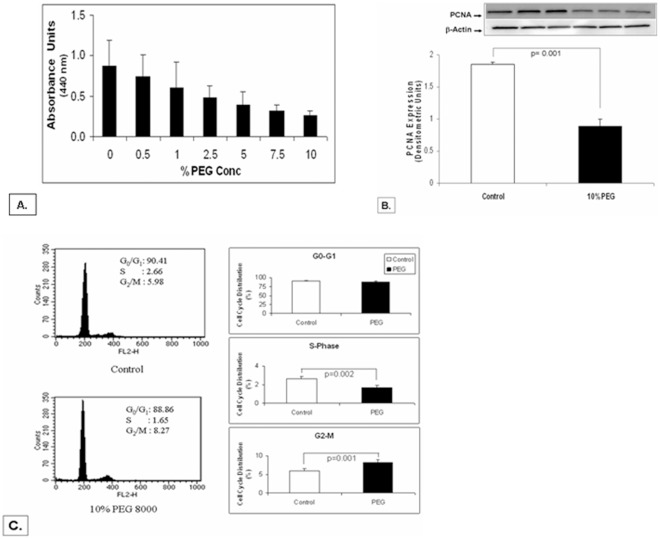
Inhibition of cellular proliferation and induction of cell cycle arrest by PEG-8000 in SCC-25 cells- SCC-25 cells were treated with different concentrations of PEG-8000 for 24 h and then assayed for proliferation using standard WST-1 assay. As shown (Figure 4A), there was a dose dependent decrease in the cell growth, with maximal decrease of 43% obtained at 10% PEG-8000. Figure 4B shows a ∼50% decrease on the expression of proliferation marker PCNA by PEG. Figure 4C shows the effect of PEG on cell cycle distribution. The 24 h PEG treated cells were stained with propidium iodide and analyzed by flow cytometry. PEG blocked cells in the S-phase (by 62%) and correspondingly increases the cells in G2-M phase (by 38%).

To understand the mechanism of PEG action on cellular proliferation in HNSCC; we used an *in vitro* cell culture model (squamous carcinoma cell line; SCC-25) to examine the effect of PEG on proliferation and cell cycle distribution. As shown in Figure 4A, the WST-1 proliferation assay revealed a dose dependent decrease in the SCC-25 cell growth when treated with PEG for 24 h, with maximal decrease of 43% obtained at 10% PEG-8000. Therefore, for subsequent experiments 24 h treatments of 10% PEG-8000 were utilized. The immunoblot results demonstrated that PEG also caused ∼ 50% decrease in proliferation marker, PCNA (Figure 4B). Cell cycle arrest is one of the important mechanisms of chemopreventive agents. To investigate the effect of PEG-8000 on the cellular distribution we performed flow cytometric analysis of PI labeled cells. Our results show that 24 h treatment of SCC-25 cells with 10% PEG-8000 resulted in a marked reduction in S-phase (proliferative) cells (62% of vehicle control; p<0.002) and a significant increase in G2-M phase cells (138% of vehicle control; p<0.001) (Figure 4C). These results clearly indicate that PEG treatment induces cell cycle arrest in hyperproliferative cells, and could therefore help restore the cellular homeostasis by modifying the altered rates of cancer cell growth and death.

### Treatment of SCC-25 Cells with PEG Decreases EGFR and Cyclin D1 and Modulates the Expression of Cell Cycle Regulatory Protein p21^cip1/waf1^

**Figure 5:**
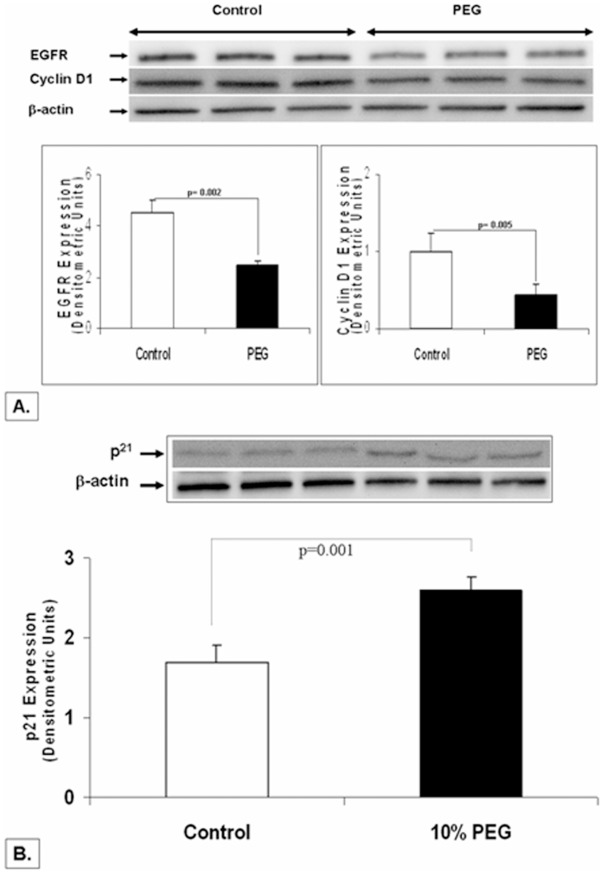
Effect of PEG-8000 on EGFR, cyclin D1 and p21 expression in SCC-25 cells-For these studies, western blot analysis was performed on lysates obtained from SCC-25 cell treated for 24 h with PEG-8000. As shown in Figure 5A, consonant with the *in vivo* experiments PEG caused a significant decrease EGFR (∼45% compared to vehicle control; p<002) and cyclin D1 (∼57% compared to control vehicle; p<0.005) expression. In a similar experimental protocol (Figure 5B), PEG-8000 caused ∼54% increase in p21 expression (p<0.001).

From our *in vivo* 4NQO rat experiments, EGFR and Cyclin D1 were the two important targets downregulated by PEG; we wanted to study if similar effects were observed in HNSCC cell lines. We first performed immunoblot experiments to assess the expression of EGFR and cyclin D1 upon 24 h treatment of PEG-8000 in SCC25 cells. Consonant with the *in vivo* experiments we found that treatment of SCC25 cells with PEG-8000 caused a significant decrease EGFR (∼45% compared to vehicle control; p<002) and cyclin D1 (∼57% compared to control vehicle; p<0.005) expression (Figure 5 A). In PEG-induced colon cancer chemoprevention, we have previously shown that cell cycle arrest induced by PEG-8000 implicates an EGFR-mediated upregulation of a cyclin-dependent kinase inhibitor, p21^cip1/waf1^ [24]. In a similar experimental protocol, we found that PEG-8000 indeed caused a ∼54% increase in p21 expression (p<0.001) in SCC25 cells (Figure 5B). Further studies are underway to characterize the upstream pathways implicated in modulation of these cell cycle regulators in SCC25 cells upon PEG treatment.

## Discussion

We herein demonstrate, for the first time, that topical application of PEG-8000 offered effective protection against oral cancer development. Our cell culture data showing anti-proliferative effects of PEG in SCC-25 cells is complemented by the decreased tumor burden in the well-validated 4-NQO-rat HNSCC model (when given in the post-initiation phase). From a mechanistic perspective, we demonstrate the downregulation of EGFR with concomitant inhibition of the cyclin D1-proliferation axis.

HNSCC should be eminently chemopreventable because the at-risk groups are readily identifiable for both primary prevention (tobacco chewing, alcohol, HPV etc) and secondary prevention. This is because HNSCC patients with just a single primary tumor have a significant risk of developing second primary tumors over the next few decades. Also, patients with oral leukoplakia (prevalence of ∼0.5%) that engender a malignant transformation rate of ∼5% per year can benefit from this chemoprevention [25]. Thus, PEG for HNSCC prevention represents a potentially clinically viable strategy.

In this regard, numerous chemopreventive agents, such as NSAIDS [26], vitamin E [27] and retinoic acid [28] have been tested against HNSCC but have failed due either to lack of efficacy or toxicity [29]. For instance, a recent randomized phase II trial, celecoxib at 100 or 200 mg twice daily was found to be ineffective in controlling oral premalignant lesions and had significant systemic toxicity such as cardiovascular side-effects [30]. Similarly, retinoids were found to offer only modest protection against development of SCC in leukoplakia patients, and with an unacceptable side-effect profile [31]. The only other agent that has shown encouraging results till to date has been green tea extract (GTE), which, while failing to achieve statistical significance in clinical response rate for oral premalignant lesions, still increased the median length of progression (27.5 to 46.4 months) [32].

The lack of compelling results with standard chemopreventive agents has led to interest in utilizing a molecularly defined candidate agent approach [33]. There have been two recent comprehensive studies evaluating the mutational landscape of HNSCC [34], [35]. These studies revealed a number of genes that are commonly altered in HNSCC. However, most of the putative early events (e.g., p53 and NOTCH1) appear to be tumor suppressor genes which are generally not “druggable” (inhibition more likely to be beneficial for proto-oncogenes). Therefore, for cancer prevention, not only do candidate targets need to be proto-oncogenes but these lesions need to be diffusely present in the oral mucosa of at-risk patients, especially in oral premalignant lesions (OPLs; mainly comprising of leukoplakia). EGFR has been shown to be a target in treatment of HNSCC with both monoclonal antibodies and small molecule inhibitors as key components of the therapeutic armamentarium. Besides, our data clearly demonstrates a profound upregulation in EGFR in the histologically normal oral/tongue mucosa in the 4NQO-treated rats. The importance of EGFR in early carcinogenesis is emphasized by a recent clinical trial which demonstrated that in most of the OPLs, EGFR upregulation (both by copy number and expression) correlated with high risk of progression to frank malignancy [36], [37]. From a chemopreventive perspective EGFR may be an important target as revealed by the mechanistic evaluation of green tea extract for cancer chemoprevention.

Our data indicates that PEG can cause a dramatic downregulation of EGFR both in cell culture and in the premalignant oral mucosa, consistent with our extensive data of PEG in colorectal carcinogenesis. Indeed, PEG is one of the most potent chemopreventive agent against colorectal cancer [8], [38]. The efficacy of PEG in colon cancer has been supported by preliminary epidemiological data [39]. From a mechanistic perspective, our laboratory has previously demonstrated that EGFR was downregulated by PEG in cell culture and in colon carcinogen (azoxymethane)-treated rat model, [13]. This effect appeared to be via membrane internalization with subsequent proteosomal degradation. The downstream consequences appear to follow the paradigm of EGFR→SNAIL→E-Cadherin→β-catenin→Tcf transcriptional regulation [13]. Importantly, the integral nature of EGFR was shown by the demonstration that EGFR knockdown (via shRNA) blunted the chemopreventive efficacy of PEG in this model of colon cancer. Studies are currently ongoing to determine the exact mechanism through which PEG internalizes EGFR. Furthermore, consistent with our current data in HNSCC, we have previously shown, in both cell culture and animal models of colon cancer, that PEG-induced EGFR downregulation suppressed proliferation potentially through cyclin D1. Furthermore, the G1→S phase cell cycle arrest observed may be mediated, at least partly, through induction of the p21^cip1/waf1^, a cyclin dependent kinase inhibitor, that may provide another modality of suppressing proliferation [24]. Both cyclin D1 and loss of p21^cip1/waf1^ have been shown to mediate the antiproliferative activity of PEG in the colon, thereby mirroring the current findings in oral cancer.

An important aspect to our HNSCC preventive strategy is the topical delivery. One of the advantages of topical delivery is decreased toxicity since there is little, if any, systemic toxicity (swish/gargle and spit would avoid enteral absorption). While it remains to be determined whether PEG delivered in this manner can adequately cover all relevant areas (including pharyngeal mucosa), topical chemopreventive agents have had some promise. For instance, ketorolac tromethamine (a nonselective COX-inhibitor) oral rinse has been shown to decrease oral prostaglandin, but not neoplasia [40]. Similarly, topical bioadhesive black raspberry gel has been shown to modulate gene expression and reduce cyclooxygenase 2 protein in human premalignant oral lesions [41].

Though topical application of PEG may potentially represent a significant advance in chemoprevention of HNSCC, there are some questions that remain to be answered. Future studies will need to answer several questions including the optimal PEG formulation. We chose PEG 8000 for these studies based on data to suggest that this was optimal molecular weight for colon carcinogenesis. However, this will need to be confirmed and/or modified for HNSCC. We will need to determine PEG formulation (concentration, viscosity etc) for better efficacy and applicability. Finally, timing for introduction of PEG treatment needs to be further assessed for achieving maximal effects. While our studies with 4-NQO were performed at a relatively late stage, this was done out of necessity to prevent confounding factors, since both the carcinogen and PEG were given topically (possibly PEG could block 4-NQO access to mucosa). Intervention earlier in tumorigenesis is likely to show a greater benefit and can be performed with other models of HNSCC (transgenic, orthotopic etc).

There are a number of limitations of this study that need to be acknowledged. First, our *in vivo* data was obtained from a well-validated HNSCC model (4-NQO-treated rat) which may not necessarily replicate the subset of human HNSCC that are related to human papilloma virus (which harbor half the mutational load of HPV negative tumors and a more muted EGFR upegulation) [42]. Second, the role and mechanism of EGFR downregulation in HNSCC remains to be fully determined. While we have demonstrated that EGFR is critical in PEG mediated chemoprevention in colon carcinogenesis, the HNSCC link is simply by analogy and requires further investigation. Furthermore, the mechanism through which PEG induced EGFR endocytosis requires elucidation in order to optimize PEG formulation.

In summary, we provide herein the proof of concept that topical PEG may be an effective chemopreventive agent against HNSCC using both cell culture and animal model. While the mechanisms remains unclear, previous work related to colorectal cancer suggests that PEG may work in HNSCC through targeting the EGFR→proliferation axis, a critical event in HNSCC. Clinically, the ability to topically prevent (via oral rinse, toothpaste, chewing gum, lozenge etc) without toxicity may potentially represent a major advance finally heralding an efficacious, cost-effective cancer prevention strategy for HNSCC.
